# D-loop haplotype diversity in Brazilian horse breeds

**DOI:** 10.1590/1678-4685-GMB-2016-0166

**Published:** 2017-08-31

**Authors:** Patrícia Ianella, Maria do Socorro Maués Albuquerque, Samuel Rezende Paiva, Andréa Alves do Egito, Leonardo Daniel Almeida, Fabiana T. P. S. Sereno, Luiz Felipe Ramos Carvalho, Arthur da Silva Mariante, Concepta Margaret McManus

**Affiliations:** 1 Embrapa Recursos Genéticos e Biotecnologia, Parque Estação Biológica, Brasília, DF, Brazil.; 2 Embrapa Gado de Corte, Campo Grande, MS, Brazil.; 3 Faculdade de Agronomia e Medicina Veterinária, Universidade de Brasília, Campus Darcy Ribeiro, Brasília, DF, Brazil.; 4 Ministério da Agricultura, Pecuária e Abastecimento, Brasília, DF, Brazil.

**Keywords:** mitochondrial DNA, genetic characterization, Equus caballus, locally adapted breeds, animal genetic resources

## Abstract

The first horses were brought to Brazil by the colonizers after 1534. Over the centuries, these animals evolved and adapted to local environmental conditions usually unsuitable for exotic breeds, thereby originating locally adapted Brazilian breeds. The present work represents the first description of maternal genetic diversity in these horse breeds based on D-loop sequences. A D-Loop HSV-I fragment of 252 bp, from 141 horses belonging to ten Brazilian breeds / genetic groups (locally adapted and specialized breeds) were analysed. Thirty-five different haplotypes belonging to 18 haplogroups were identified with 33 polymorphic sites. Haplotype diversity (varying from 0.20 to 0.96) and nucleotide diversity (varying from 0.0039 to 0.0239) was lower for locally adapted than for specialized breeds, with the same pattern observed for F_ST_ values. Haplogroups identified in Brazilian breeds are in agreement with previous findings in South American samples. The low variability observed mainly in locally adapted breeds, indicates that, to ensure conservation of these breeds, careful reproductive management is needed. Additional genetic characterization studies are required to support accurate decision-making.

Horses have played an important role in shaping human civilization and their domestication occurred from several wild populations 4.000 to 6.000 years ago (Vaughan *et al.*, 2010; Outram *et al.*, 2009). This species was introduced in South America by European conquerors during the 16th century, and the development of current American horse breeds has been based on the ones from the Iberian Peninsula (Jimenez *et al.*, 2012; Cortés *et al.*, 2017). As with all domesticated animals introduced during this period, horses were widely dispersed in this new environment, becoming adapted to different conditions (Mirol *et al.*, 2002). The first horses were brought to Brazil by the colonizers after 1534 (Primo, 2004). Over the centuries these animals evolved and adapted to conditions usually unsuitable to exotic breeds, including local environmental (high temperatures, long periods of drought), sanitary (vector-born disease) and management systems found in Brazil, originating Brazilian breeds also known as “locally adapted” or “creoles”. The main locally adapted Brazilian genetic groups include the breeds Campeira (Santa Catarina), Creole (Rio Grande do Sul), Lavradeira (Roraima), Pantaneira (Pantanal - Mato Grosso), Mangalarga (Minas Gerais and São Paulo), Marajoara (Marajó Island - Pará), as well as smaller animals such as the Puruca pony (Pará) and the genetic group Baixadeiro (Maranhão). More recently, to improve conformation, and increase stature, these breeds were crossed with Arab or English Thoroughbred breeds (Beck, 1985). Considering the importance of these breeds/genetic groups in their respective geographic regions and set the hypothesis that they are facing a genetic erosion, the main objective of this study is to quantify the genetic distribution of mtDNA haplotypes of the locally adapted breeds and genetic groups from Brazil and compare this with the information already generated for specialized breeds.

Genetic characterization of cryopreserved and live animals is an important approach for orientating conservation strategies (Solis *et al.*, 2005). Mitochondrial DNA (mtDNA) sequencing has imprinted biogeographic and phylogenetic perspectives on intra- and inter-species genetic structure. The displacement loop hyper-variable region of mtDNA (D-loop) is useful for population and evolutionary studies because of its high level of sequence variation, in addition to a lack of recombination and maternal heritance. D-loop polymorphisms have been used to understand the origin and genetic diversity of horses from Italy (Bigi *et al.*, 2014), Iran (Moridi *et al.*, 2013), India (Devi and Ghosh, 2013), China (Zhang *et al.*, 2012), Colombia (Jimenez *et al.*, 2012), Croatia (Ivankovic *et al.*, 2009) and Lithuania (Cothran *et al.*, 2005), as well as Lusitano (Hill *et al.*, 2002; Lopes *et al.*, 2005) and Arabian horses (Khanshour and Cothran, 2013). These studies have also been used to infer on horse phylogeography and evolution (Jansen *et al.*, 2002; Mirol *et al.*, 2002; McGahern *et al.*, 2006; Cieslak *et al.*, 2010; Achilli *et al.*, 2012; Jimenez *et al.*, 2012; Devi and Ghosh, 2013). According to Mirol *et al.* (2002), knowledge of South American breeds is important for conservation genetics of domestic horses, as New World varieties are, probably, closer to historical horses than those found currently on the Iberian Peninsula, which have been crossed with other breeds over the last 500 years. In spite of the wide range of Brazilian locally adapted breeds, few studies have been carried out with microsatellite data to investigate their genetic diversity and establish relationships between them. Most of the data indicated loss if variability for Brazilian locally adapted breeds (Reis *et al.*, 2008; De Assis *et al.*, 2009; Silva *et al.*, 2012; Pires *et al.*, 2016), except in the Pantaneiro breed, for which relatively high levels of heterozygosity were found (Giacomoni *et al.*, 2008; Sereno *et al.*, 2008).

To investigate mtDNA diversity in specialized (commercial breeds) and locally adapted breeds (creoles) of horses in Brazil, 141 animals belonging to 10 locally adapted and commercial Brazilian breeds/genetic groups were analysed (Table 1). Analyses of sequences from the HVS-I region of the mitochondrial D-Loop sequence (Ishida *et al.*, 2009) were carried out. PCR reactions were performed in a 20 μL final volume containing: 9 ng of DNA, 0.25 μm of each primer, 0.20 mM dNTP, 1X PCR buffer (1M Tris HCL, pH 8.4 100 mM, KCL 500 mM), 2.5 mM MgCl_2_, 1U *Taq* polymerase. Amplifications started with an initial denaturation step of 94 °C for 5 min, followed by 35 cycles of 1 min at 94 °C, 59 °C for 1 min, 72 °C for 1 min, and a final extension of 5 min at 72 °C. PCR products were purified with Exo-SAP enzyme and sequenced using a BigDye v3.1 sequencing kit (Applied Biosystems, Foster City, CA, USA) and a DNA sequencer ABI 3130 (Applied Biosystems) according to the manufacturer's manual.

**Table 1:** Estimated haplotype and nucleotide diversity from the d-loop region of mtDNA, in locally adapted and specialized Brazilian horse breeds.

Breed	Number of animals	Number of haplotypes	Haplotype diversity	Nucleotide diversity	F_ST_
Baixadeira (EBA)	10	3	0.600 ± 0.131	0.01667 ± 0.00637	0.015
Campeira (ECA)	10	3	0.600 ± 0.132	0.00899 ± 0.00462	0.008
Lavradeira (ELV)	10	2	0.200 ± 0.154	0.00397 ± 0.00314	0.004
Marajoara (EMA)	10	4	0.778 ± 0.091	0.01437 ± 0.00521	0.013
Puruca (EPU)	10	5	0.756 ± 0.130	0.01120 ± 0.00579	0.009
Pantaneira (EPA)	43	16	0.909 ± 0.025	0.01787 ± 0.00614	0.018
Criolla (ECR)	11	5	0.733 ± 0.155	0.00661 ± 0.00361	0.002
Arab (EAR)	12	9	0.962 ± 0.040	0.02260 ± 0.01149	0.023
Mangalarga (EML)	9	7	0.944 ± 0.070	0.02116 ± 0.00859	0.020
English Thoroughbred (ETB)	16	12	0.942 ± 0.048	0.02394 ± 0.01198	0.025
Total	141	33	0.9250.011	0.01942 ± 0.00672	0.022

Fst – Fixation Index

The sequences obtained were aligned with the reference sequence (GenBank Accession number NC_001640) using SeqScape v2.6, as well as other sequences deposited in GenBank. After edition, the sequences (252 bp/animal) were analyzed in MEGA V.3.1 (Kumar *et al.*, 2004) and DNASP V.4.5 (Rozas *et al.*, 2003) programs to determine the number of haplotypes. Haplotypes were classified in haplogroups according to the nomenclature established by Achilli *et al.* (2012). The analysis of Molecular variance (AMOVA) and *Fst* distances between pairs of breeds were carried out using the Arlequin V. 3.0 (Excoffier *et al.*, 2005) program. Free software NETWORK 5.0.0.1 (http://www.fluxus-engineering.com) was used for calculation of the median joining network (Bandelt *et al.*, 1999) of all haplotypes.

Thirty-six different haplotypes were identified with 33 polymorphic sites (Table 2). Of the 18 haplogroups found by Achilli *et al.* (2012), nine (A, B, H, I, L, M, N, O'P' and Q) were found in the Brazilian samples, and seven (except H and O'P') were identified in the locally adapted breeds/genetic groups (Table 2 and Figures 1 and 2). Achilli *et al.* (2012) have also identified these same haplogroups in South American samples, except for the H haplogroup, a rare type observed only in Asia and Europe (here observed in one EAR and one ETB). These authors have proposed a new nomenclature, constituting 18 haplogroups observed in ancient and modern horses worldwide. All these haplogroups are present in Asian horses and 17 in European horses (except for F – present only in Przewalskis horse, an Asian subspecies wild horse).

**Figure 1:**
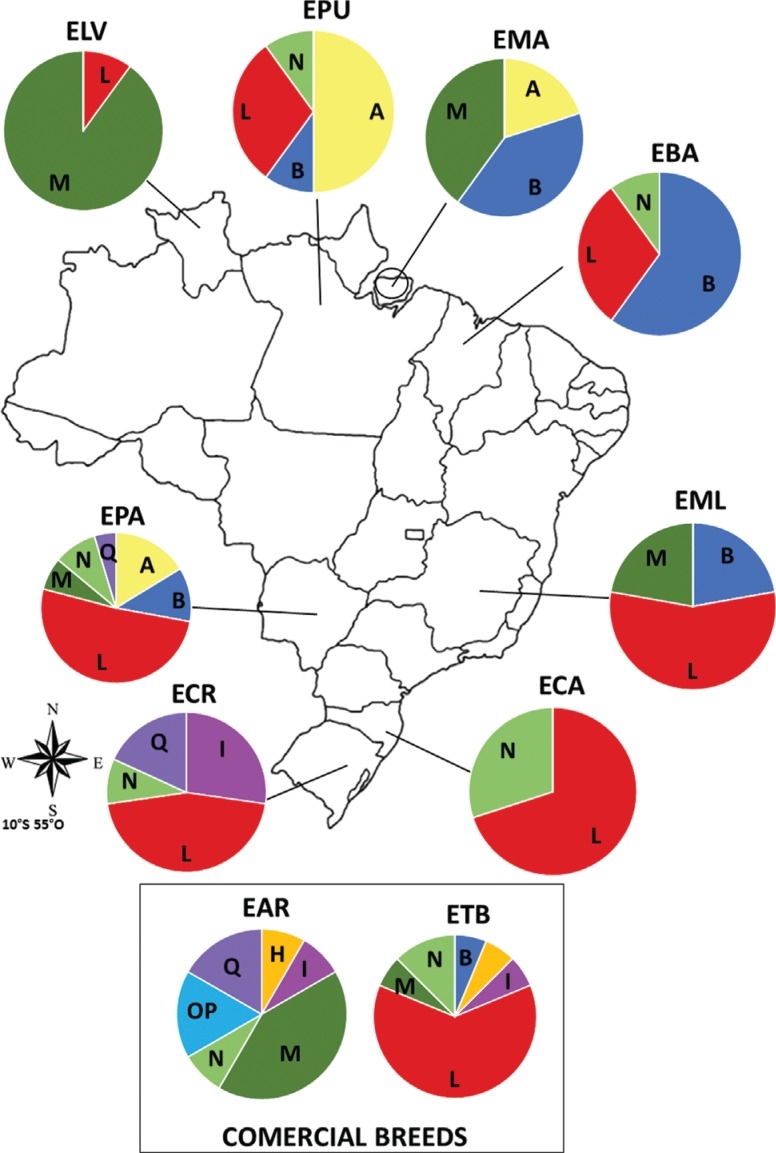
Haplogroup frequency in Brazilian horse breeds. Haplogroup classification is according to Achilli *et al.* (2012). Horse Breeds: ELV - Lavradeira; EPU - Puruca; EMA - Marajoara; EBA - Baixadeira; EPA - Pantaneira; ECR - Crioula; EML - Manga Larga; ECA - Campeira; EAR - Árabe; ETB - English Thoroughbreed.

**Figure 2:**
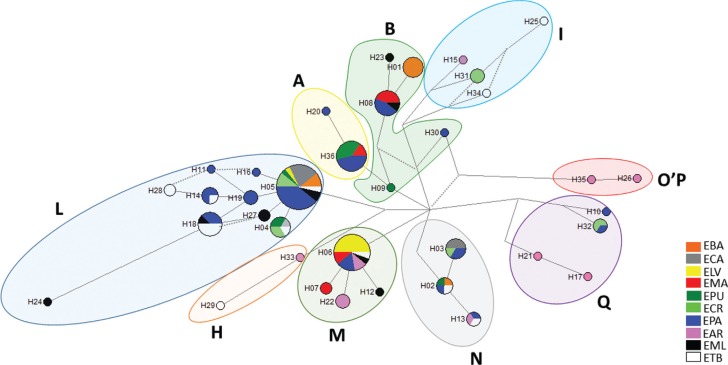
Median-joining network analysis of 36 haplotypes from 141 Brazilian horse mitochondrial DNA. Circles represent the haplotypes, and their sizes are proportional to the frequency. Colored shapes represent haplogroup classification according to Achilli *et al.* (2012). Horse Breeds: ELV - Lavradeira; EPU - Puruca; EMA - Marajoara; EBA - Baixadeira; EPA - Pantaneira; ECR - Crioula; EML - Manga Larga; ECA - Campeira; EAR - Árabe; ETB - English Thoroughbreed.

**Table 2:** Haplotype (H) list showing nucleotide substitutions observed in mitochondrial DNA D-Loop (252 bp) in Brazilian equine breeds, the position of polymorphic nucleotide sites and number of animals observed in each breed analyzed.

Position of nucleotide substitution relative to GenBank NC_001640 as Reference Sequence																																			Breed									
	Haplogroup*	15526	15534	15538	15540	15555	15580	15582	15584	15585	15596	15597	15601	15602	15603	15604	15608	15611	15617	15649	15650	15659	15666	15667	15703	15709	15718	15720	15723	15726	15740	15743	15745	15755	EBA	ECA	ELV	EMA	EPU	ECR	EPA	EAR	EML	ETB
NC_001640 - H36	A	T	C	A	A	C	T	T	C	G	A	A	T	C	T	G	C	G	T	A	A	T	G	A	T	C	C	G	C	G	A	A	C	G				2	5		6			
H1	B	.	.	.	.	.	.	.	.	A	.	.	.	.	.	.	.	.	.	.	G	.	A	.	.	.	.	A	.	.	.	.	.	.	6									
H2	N	.	.	.	.	.	.	.	.	A	.	.	C	T	.	.	.	.	.	.	.	.	.	.	.	.	.	A	.	.	.	.	.	.	1				1		1			1
H3	N	.	.	.	.	.	.	.	.	.	.	.	C	T	.	.	.	.	.	.	.	.	.	.	.	.	.	A	.	.	.	.	.	.		3				1	2			
H4	L	.	T	.	.	.	.	.	.	.	.	.	.	T	C	.	.	.	.	G	.	.	.	.	.	.	.	.	.	.	.	.	.	.		1			2	2				1
H5	L	.	T	.	.	.	.	.	.	.	.	.	.	T	C	.	.	.	.	G	.	.	.	.	.	.	.	A	.	.	.	.	.	.	3	6	1		1	3	11		2	1
H6	M	.	.	.	.	.	.	.	.	.	.	.	.	T	.	.	.	.	C	.	.	C	.	.	.	.	.	A	.	.	.	.	.	.			9	2			3	2	1	1
H7	M	.	.	.	.	.	.	.	.	.	.	.	.	T	.	.	.	.	C	.	.	C	.	.	.	.	.	.	.	.	.	.	.	.				2						
H8	B	.	.	.	.	.	.	.	.	.	.	.	.	.	.	.	.	.	.	.	G	.	A	.	.	.	.	A	.	.	.	.	.	.				4			4		1	
H9	B	.	.	.	.	.	.	.	.	.	.	.	.	.	.	.	.	.	.	.	.	.	.	.	.	.	.	A	.	.	.	.	.	.					1					
H10	Q	.	.	.	.	.	.	.	.	.	.	.	.	T	.	.	.	.	.	.	.	.	.	.	C	.	.	A	T	.	G	.	.	A							1			
H11	L	.	T	.	.	.	.	.	.	A	.	.	.	T	C	.	.	.	.	G	.	.	.	.	.	.	.	A	.	.	.	.	.	A							1			
H12	M	.	.	.	.	.	.	.	.	.	.	.	.	T	.	.	.	.	C	.	.	C	.	.	.	.	.	A	.	.	.	.	.	A									1	
H13	N	.	.	.	.	.	.	.	.	A	.	.	C	T	.	.	.	.	.	.	.	.	.	.	.	.	.	A	.	.	.	.	.	A							1	1		1
H14	L	.	T	.	.	.	.	.	.	A	.	.	.	.	C	.	.	.	.	G	.	.	.	.	.	.	.	A	.	.	.	.	.	.							3			1
H15	I	.	.	G	.	.	.	.	.	.	G	.	.	T	.	.	.	.	.	.	G	.	.	.	.	T	.	A	.	.	.	.	.	.								1		
H16	L	.	T	.	.	.	.	.	.	.	.	.	.	T	C	.	.	.	.	G	.	.	.	.	.	.	.	A	.	.	.	.	.	A							1			
H17	Q	.	.	.	.	.	.	.	.	A	.	.	.	T	.	A	.	.	.	.	.	.	.	.	C	.	.	A	.	A	G	.	.	.								1		
H18	L	.	T	.	.	.	.	.	.	A	.	.	.	T	C	A	.	.	.	G	.	.	.	.	.	.	.	A	.	.	.	.	.	.							3		1	4
H19	L	.	T	.	.	.	.	.	.	A	.	.	.	T	C	.	.	.	.	G	.	.	.	.	.	.	.	A	.	.	.	.	.	.							3			
H20	A	.	.	.	.	.	.	.	.	.	.	.	.	.	.	.	.	.	.	.	.	.	.	.	.	.	.	.	.	.	.	.	.	A							1			
H21	Q	.	.	.	.	.	.	.	.	A	.	.	.	T	.	A	.	.	.	.	.	.	.	.	C	.	.	A	.	.	G	.	.	.								1		
H22	M	.	.	.	.	.	.	.	.	A	.	.	.	T	.	.	.	.	C	.	.	C	.	.	.	.	.	A	.	.	.	.	.	.								3		
H23	B	.	.	.	.	.	.	.	.	.	.	.	.	.	.	.	.	.	.	.	G	.	A	.	.	.	.	A	.	.	.	.	.	A									1	
H24	L	.	T	.	.	T	G	G	.	A	.	.	.	T	C	A	A	A	.	G	.	.	.	.	.	.	.	A	.	.	.	T	T	.										1
H25	I	.	.	G	.	.	.	.	T	A	.	.	.	T	.	.	.	.	.	.	G	.	.	.	.	T	.	A	.	.	.	.	.	A										1
H26	OP	.	.	.	.	.	.	.	.	.	.	G	.	T	.	A	.	.	.	.	.	.	.	G	C	.	.	A	.	.	.	.	.	A								1		
H27	L	.	T	.	.	.	.	.	.	.	.	.	.	T	C	A	.	.	.	G	.	.	.	.	.	.	.	A	.	.	.	.	.	.									2	
H28	L	.	T	.	.	.	.	.	.	A	.	.	.	.	C	.	.	.	.	G	.	.	.	.	.	.	.	A	.	.	.	.	.	A										2
H29	H	C	.	.	G	.	.	.	.	A	.	.	.	T	.	.	.	.	.	G	.	.	.	.	.	.	T	A	.	.	.	.	.	.										1
H30	B	.	.	.	.	.	.	.	.	.	.	G	.	T	.	.	.	.	.	.	G	.	.	.	.	.	.	A	.	.	.	.	.	.							1			
H31	I	.	.	G	.	.	.	.	.	A	.	.	.	T	.	.	.	.	.	.	G	.	.	.	.	T	.	A	.	.	.	.	.	.						3				
H32	Q	.	.	.	.	.	.	.	.	.	.	.	.	T	.	.	.	.	.	.	.	.	.	.	C	.	.	A	T	A	G	.	.	.						2	1			
H33	H	C	.	.	.	.	.	.	.	A	.	.	.	T	.	.	.	.	.	G	.	.	.	.	.	.	T	A	.	.	.	.	.	.								1		
H34	B	.	.	G	.	.	.	.	T	A	.	.	.	T	.	.	.	.	.	.	G	.	.	.	.	.	.	A	.	.	.	.	.	.										1
H35	OP	.	.	.	.	.	.	.	.	.	.	G	.	T	.	A	.	.	.	.	.	.	.	G	C	.	.	A	.	.	.	.	.	.								1		

EBA – Baixadeira; ECA – Campeira; ELV – Lavradeira; EMA – Marajoara; EPU – Puruca; ECR – Criolla; EPA – Pantaneira; EAR – Arab, EML – Mangalarga; ETB- English Thoroughbred.

* Haplogroups defined by mtDNA nomenclature according to (Achilli *et al.*, 2012).

The majority of mtDNA sequences from Brazilian locally adapted breeds belong to haplogroup L (Haplotype H5, 28 individuals, Figure 2) although this haplogroup was not found in locally adapted Marajoara (EMA) and specialized Arab (EAR) breeds (Table 1). The EMA breed is found on an island (Marajó) off the coast of Pará state in northern Brazil. Given its isolation, there are less opportunities for crossbreeding moreover the island has a largely inhospitable terrain, such as flooded marshlands (Reis *et al.*, 2008) with high indices of tropical diseases, such as IEA (Equine infectious anemia). The O'P' haplogroup was observed only in the Arab breed. The number of haplotypes per breed varied between two (Lavradeira-ELA) and 16 (Pantaneira-EPA) (Table 2). Twenty-five unique haplotypes were observed in the locally adapted breeds/genetic groups: EMA and Creole (ECR) Baixadeira (EBA), showing one each, and EPA showing seven unique haplotypes. Specialized breeds, such as EAR and Thoroughbred (ETB) showed seven and five unique haplotypes, respectively (Table 1). To better understand how haplotypes are distributed across the breeds, a network analysis for the 36 haplotypes was carried out (Figure 2). This analysis corroborates with the haplogroup classification, showing the separation of the haplotypes into haplogroups according to the branches of the network.

According to Reis *et al.* (2008), Marajora (EMA) had slightly higher diversity than Puruca (EPU), as also observed in this study. Such a finding may be due to a larger population and broader founder base. According to Reis *et al.* (2008), the Marajoara horses was derived from Portuguese horses from Cabo Verde, introduced into the Marajó archipelago at the beginning of the 18th century, while the Puruca pony was derived from nine Shetland ponies imported from France at the end of the 19th century.

The haplotype diversity index (Table 1) was highest for EAR (0.962) and lowest for ELV (0.200). EPA showed the highest haplotype diversity among the locally adapted breeds (0.900). The lowest nucleotide diversity was observed as well in ELV (0.00397) and the highest in EPA (0.1787) among the locally adapted breeds. The specialized breed ETB showed the highest nucleotide diversity index among the breeds analyzed (0.02394). The observed haplotype and nucleotide diversity average in this study was lower than that recorded in Italian (Bigi *et al.*, 2014), Indian (Devi and Ghosh, 2013), Colombian Creole (Jimenez *et al.*, 2012) and Chinese horses (Zhang *et al.*, 2012). The ELV showed the lowest haplotype and nucleotide diversity index, and the individuals of this studied population grouped only in two haplogroups, which may be related to its geographic isolation. EPA showed the highest diversity indices among local breeds., and it has mtDNA classified in six haplogroups with three shared haplotypes H13, H14 and H18 with specialized breeds. These findngs may be due to the use of the breed in different crosses with specialized breeds in an attempt to increase the physical stature and improve performance. In addition, it is important to note that EPA is the most prominent locally adapted breed in Brazil, and it has a growing trend in the internal market. Such high genetic variability was also observed in other studies using RAPD (Egito *et al.*, 2007) and microsatellite markers (Giacomoni *et al.*, 2008; Sereno *et al.*, 2008).

Values of breed differentiation (*F_ST_*) were expectedly low, varying between breeds (from 0.002 in ECR to 0.025 in ETB, mean 0.02), suggesting a low population structure level (Table 1). Silva *et al.* (2012), using microsatellites to characterize locally adapted horse breeds, demonstrated loss of genetic variability in Campeira, Lavradeira, Baixadeira and Mangalarga Marchador horses in Brazil. Similar results were described using microsatellite analyses in Marajoara and Puruca (Reis *et al.*, 2008) and Mangalarga breeds (De Assis *et al.*, 2009). This highlights the need for conservation efforts for these breeds, especially those found in highly challenging environments where tropical diseases can devastate local populations and severely affect subsistence farmers who depend on these animals for their living.

Haplogroups identified in Brazilian breeds are in concordance with previously findings in South American samples, as described in the literature. Despite the important adaptive characteristics retained in these breeds altogether, a loss of genetic variability can be seen. Locally adapted breeds, except Pantaneira, tend to show lower haplotype diversity than commercial breeds. This may be associated with their history of geographical isolation, such as for Lavradeira and Marajoara, local adaptations to stressful environmental conditions (most locally adapted breeds), or long term breeding strategies, such as with the Crioula, for which a herd book has been maintained for almost 100 years (Maciel *et al.*, 2014).

Genetic diversity is essential to maintain current production needs in several environments, allowing sustained genetic improvement and facilitating faster adaptation to changing breeding objectives (Notter, 1999). Careful selection of animals for breeding and conservation should be carried out to ensure that variability is maintained within these populations. Paiva *et al.* (2011) showed that the integration of different genetic strategies is useful in conservation programs to provide different types of information and so optimize the selection of animals for breeding purposes. The mtDNA haplotypes, along with nuclear molecular markers, may be important as an additional criterion for genetic management of animals in conservation nuclei, as well as a proxy to help the germplasm collection to be deposited in the national gene bank.
